# The emerging role of acceptance and commitment therapy as a way to treat trauma and stressor related disorders

**DOI:** 10.1192/bjo.2021.770

**Published:** 2021-06-18

**Authors:** Gisela Simões, Rita Silva

**Affiliations:** Department of Psychiatry and Mental Health of Baixo Vouga Hospital Centre, EPE

## Abstract

**Aims:**

The aim of this work is to gather and evaluate scientific evidence about the clinical effects of Acceptance and Commitment Therapy (ACT) in the treatment of patients with trauma-related Post-Traumatic Stress Disorder (PTSD).

**Method:**

A literature search was conducted on PubMed platform, starting from the following MeSH terms: “Acceptance and Commitment Therapy”, “Trauma and Stressor Related Disorders”, “Psychological Trauma”. Studies obtained were analysed, corresponding to investigations based on an adult population with trauma and stressor related disorders.

**Result:**

The search provided 13 results, of which 12 met the defined criteria. Different types of studies with variable samples were considered, including randomised clinical trials, longitudinal observational studies, narrative reviews and an analysis of case reports.

Globally, ACT has been showing a crescent role in the treatment of individuals with trauma histories by enhancing positive outcomes and by being associated with greater psychological flexibility. It is increasingly considered to be well-suited to the treatment of trauma by targeting avoidance, coping strategies with emotional disengagement and persistent dissociation, aspects associated with greater PTSD symptom severity and related psychopathology.

Furthermore, research suggests that acceptance-based treatments are helpful in promoting emotional, behavioural, and neural changes in psychological disorders characterised by disgust, shame and guilt that commonly co-occur with PTSD.

Among the various exposure factors, we found a growing production of recent literature in which ACT has been applied in the context of oncology life-threatening settings, demonstrating significant improvements in symptoms and quality of life, as well as reductions in emotional disturbances, physical pain and traumatic responses.

However, little is known about implementation and results of ACT in situations of trauma and psychiatric comorbidities. Data suggest that, when applied to individuals with psychosis and history of trauma, there is an improvement in overall severity and anxiety symptoms, emotion regulation strategies and a greater sense of engagement in care; nevertheless, reduction of specific trauma symptoms remains controversial. More mention is made about the growth of literature evaluating the application of ACT as a conjunctial therapeutic method for trauma and simultaneous addictive disorders.

**Conclusion:**

Overall, despite limited published research currently available, some evidence starts to support ACT's promising role as an effective psychotherapeutic approach to trauma and stressor related disorders. Its application in situations where organic diseases represent stress factors has been growing. Future research should focus on clarifying the role of ACT in psychiatric comorbidity scenarios, allowing this psychotherapy to help individuals find a meaningful and valuable life beyond trauma.

